# THE BNIP3 TRIAD: MITOCHONDRIA, LYSOSOMES AND INFLAMMATION IN HEALTHY MUSCLE AGING

**DOI:** 10.1080/27694127.2022.2082202

**Published:** 2022-06-05

**Authors:** Andrea Irazoki, Antonio Zorzano, David Sebastian

**Affiliations:** a Institute for Research in Biomedicine (IRB Barcelona), The Barcelona Institute of Science and Technology, Baldiri Reixac, 10-12, Barcelona, 08028 Spain; b Departament de Bioquímica i Biomedicina Molecular, Facultat de Biologia, Universitat de Barcelona, Barcelona, 08028 Spain; c Centro de Investigación Biomédica en Red de Diabetes y Enfermedades Metabólicas Asociadas (CIBERDEM), Instituto de Salud Carlos III, Madrid, Spain

**Keywords:** Aging, inflammation, lysosome, mitochondria, mitophagy, muscle, sarcopenia

## Abstract

During aging, skeletal muscle undergoes a loss of mass, strength and function, encompassed in the biological process termed sarcopenia. Given demographic aging, it is essential to comprehend the molecular determinants resulting in age-related sarcopenia. Here we studied the role of the mitophagy adaptor BNIP3 in mitochondrial homeostasis and muscle during aging. Our findings reveal that BNIP3 orchestrates mitochondrial and lysosomal function to mitigate muscle inflammation and atrophy during aging.

Age-associated sarcopenia is a fundamental contributor to disability and loss of autonomy in the elderly, thus compromising quality of life. A contributing factor to this process is the presence of chronic inflammation, which has been described in diverse epidemiological studies. Muscle aging is also characterized by mitochondrial dysfunction and defective removal of damaged mitochondria (mitophagy) and, interestingly, recent evidence points to direct links between these two events and the triggering of inflammatory responses. However, the intracellular systems orchestrating chronic muscle inflammation and sarcopenia in the context of aging have not been explored to date.

To address this conundrum, we evaluated the participation of the mitophagy protein BNIP3 (BCL2/adenovirus E1B interacting protein 3) in governing mitochondrial function and mitophagy and in mitigating inflammation. Although BNIP3 has been extensively studied upon several metabolic interventions, its contribution to the maintenance of mitochondrial homeostasis in skeletal muscle in the context of aging remained unaddressed. To tackle this knowledge gap, here we used a range of tools, including cultured myotubes, aged mice and human muscle biopsies from subjects of different ages, to obtain a comprehensive view of the molecular and physiological events that occur during muscle aging and that link BNIP3 to mitochondrial dysfunction, defective mitophagy, inflammation, and sarcopenia [1].

Characterization of mitochondrial function in BNIP3-knock down (KD) myotubes revealed that BNIP3 is key in maintaining mitochondrial health. A decrease in BNIP3 disrupts mitophagy, causing mitochondrial dysfunction. The accumulation of autolysosomes in BNIP3 KD myotubes led us to study the role of this protein in lysosomal function. Strikingly, we observed that BNIP3 KD myotubes show altered lysosomal mass and acidity, along with decreased activity of lysosomal proteases. These observations thus suggest that BNIP3 is key to maintaining not only mitochondrial function but also lysosomal function. In fact, thorough analysis of the subcellular location of BNIP3 in cultured muscle cells and skeletal muscle from mice revealed that BNIP3 localizes not only in mitochondria but also in the membranes of lysosomes, thereby supporting the notion that it exerts a function in lysosomes beyond its role as a mitochondrial adapter in mitophagy. Thus, our data show that BNIP3 tunes both mitochondrial and lysosomal function, and this orchestration allows the maintenance of mitophagic activity and, hence, cellular homeostasis.

To assess the impact of altered mitochondrial and lysosomal function induced by BNIP3 deficiency, and given the increasing evidence linking mitochondrial and mitophagy dysregulation to inflammation, we examined the inflammatory state of BNIP3-deficient myotubes. Our data show upregulation of inflammatory genes, leading to the secretion of pro-inflammatory cytokines upon BNIP3 downregulation. In fact, given the reduced degradation of damaged mitochondria in these conditions, mitochondrial DNA (mtDNA) accumulates in lysosomes, resulting in an enhanced encounter of mtDNA with the DNA sensor TLR9 (toll-like receptor 9). From the mechanistic perspective, we demonstrate that TLR9 blockade in BNIP3-deficient myotubes rescues the inflammatory phenotype, thus establishing a molecular link between the dual role of BNIP3 in mitochondrial and lysosomal homeostasis and the activation of inflammatory pathways.

During aging, healthy mice show a progressive upregulation of BNIP3 expression in skeletal muscle. This observation would infer that BNIP3 is modulated in an age-associated manner. To address the physiological implication of the newly described mechanism in the context of muscle aging, we characterized the muscle health of aged mice upon repression of BNIP3 in skeletal muscles. The muscle of these animals shows exacerbated muscle inflammation compared to that of contralateral controls. This exacerbation is accompanied by enhanced muscle atrophy, characterized by a decreased cross-sectional area (CSA), upregulated atrophy-related gene/atrogene expression, and increased denervation. Our results thus reveal that the molecular mechanisms that link BNIP3 deficiency to the activation of inflammation mediated by the accumulation of damaged mitochondria also participate in the development of age-associated muscle atrophy in mice (Fig.1).

**Figure 1.**
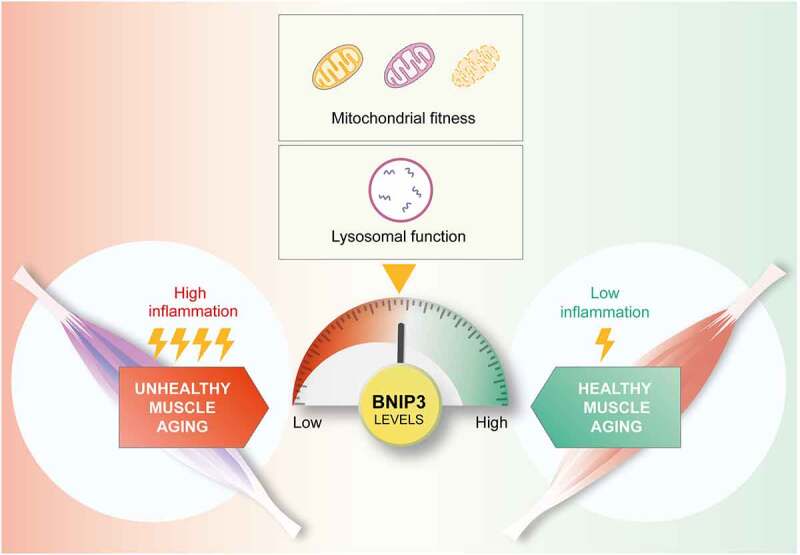
BNIP3 tunes mitochondrial and lysosomal homeostasis to promote healthy aging. Muscle BNIP3 increases during aging in mice and humans, sustaining mitochondrial and lysosomal function, which represents an adaptive mechanism that confers resistance to age-induced inflammation and muscle atrophy.

To provide further physiological support of these findings, we assessed BNIP3 expression and muscle health parameters in human muscle biopsies from young and aged subjects. We first observed upregulated BNIP3 expression associated with muscle aging, which suggests that age-induced BNIP3 modulation is conserved in mice and humans. Importantly, when stratifying aged subjects into low and high BNIP3 expressers, we detected a clear association between BNIP3, inflammation and the probability of developing sarcopenia and other comorbidities, as measured by the Charlson comorbidity index (CCI). Indeed, whereas low BNIP3 expressers show enhanced inflammation and CCI, high expressers exhibit remarkable reductions in these two parameters. These data suggest that the age-induced modulation of BNIP3 levels in the muscle acts as a preventive mechanism to mitigate unhealthy muscle aging. This notion was confirmed by the finding that aged subjects with low muscle BNIP3 expression present worsened muscle aging characterized by increased inflammation and likelihood of developing sarcopenia. These observations highlight the physiological relevance of the molecular mechanism linking BNIP3 expression to mitochondrial and lysosomal dysfunction and inflammation in the context of muscle aging.

In summary, our findings reveal that BNIP3 orchestrates mitochondrial and lysosomal function to promote the correct degradation of defective mitochondria in muscle cells. Disruption of this balance results in the activation of inflammatory signals, which have a negative impact on muscle aging. Indeed, reduced BNIP3 expression in both human and mouse muscle is associated with inflammation and impaired muscle health, thereby suggesting that the age-related increase in BNIP3 levels is essential for healthy muscle aging. Overall, our data reveal a new molecular mechanism that links mitochondrial dysfunction and the triggering of inflammation, as well as the importance of this mechanism in the context of muscle aging.
